# The impact of adapted exercises in basketball on the perception of the difficulty and physical enjoyment of students with overweight

**DOI:** 10.1016/j.heliyon.2024.e29190

**Published:** 2024-04-03

**Authors:** Oumayma Slimi, Antonella Muscella, Santo Marsigliante, Mourad Bahloul

**Affiliations:** a High Institute of Sport and Physical Education, University of Sfax, Sfax, 3000, Tunisia; b Research Laboratory: “Education, Motricité, Sport et Santé”, EM2S, LR19JS01, High Institute of Sport and Physical Education of Sfax, University of Sfax, Sfax, 3000, Tunisia; c Department of Biological and Environmental Sciences and Technologies (Di.S.Te.B.A.), University of Salento, Lecce, Italy; d Higher Institute of Education and Continuing Training, Virtual University of Tunis, Tunis, Tunisia; e Higher Institute of Business Administration of Sfax, University of Sfax, Sfax, Tunisia

**Keywords:** Basketball, Adapting, Perception, Overweight: difficulty, Physical enjoyment

## Abstract

The purpose of this study was to investigate the effect of the adapted basketball cycle on the perceived level of difficulty and physical enjoyment in girls and boys with overweight. Sixty-six students with overweight (aged 16–18 years) were randomly assigned to an experimental group (EG, n = 32 including 20 boys and 12 girls) and a control group (CG, n = 34; including 21 boys and 13 girls). Statistical analysis also showed that the levels of perceived difficulty decreased significantly after of seven-week of the adapted basketball cycle in both boys (Δ% = - 0.27; p < 0.001; and girls (Δ% = - 0.36; p < 0.001). There was no significant difference in students who participated in the traditional basketball cycle.

A physical activity enjoyment Scale revealed that in girls, the level of enjoyment increased significantly (Δ% = + 0.27; P < 0.001) after an adapted basketball cycle. There was no significant change in physical enjoyment in boys EG (P = 0.808) and participants in the control groups. These results push us to opt more for adapted and motivational learning situations to make the teaching-learning process better, in students with overweight.

## 1 Introduction

Childhood and adolescent obesity are a serious public health problems widespread not only in developed countries but also in urban areas of middle- and low-income countries [1,2]. Unfortunately, overweight and, in particular, obesity are correlated to various risk factors for cardiovascular diseases, hypertension, type 2 diabetes and other diseases [3]. Obesity corresponds to an excess of fat mass in the body due to poor dietary habits associated with an overly sedentary lifestyle and physical inactivity [4,5].

Thus, both sedentary behaviors and physical inactivity strengthen the risk of developing overweight and obesity already during childhood [6,7]. In addition, scarce physical activity and childhood obesity are related, as high weight negatively affects motor coordination performance for age and sex [6]. Obese girls face poor mental well-being, body shaming and unmotivated behavior [8], and interventions of adapted physical activity reduce social to overweight girls' distance [9].

This scarce development of motor skills [6,10] is the main cause of physical condition decline, and as soon as their body weight is engaged in a physical test, they showed less performance including cardiorespiratory fitness, muscular endurance, balance, speed, or [11,12]. It is well demonstrated that the motor skills of overweight or obese children are lower than those of normal weight children [11,13]. Furthermore, the agility, resistance, power of the lower limbs, speed and balance of obese children are significantly lower than those of children with normal weight [11,14]. Fogelholm et al. [15] indicated that young people with obesity showed lower physical condition, in particular, because of their low cardiorespiratory fitness, other physical qualities can be compensated by a high level of practice.

Furthermore, obese and sedentary children demonstrate lower psychological well-being than physically active normal-weight children [16] and, as adults, tend to remain overweight, together with poor mental health [17].

Therefore, many studies have suggested that encouraging children to follow physical activity and diet quality programs could prevent and improve the problem of childhood with overweight or obesity [[18], [19], [20], [21]].

School is a privileged place for learning to live together for all children, adolescents, and young adults, regardless of their differences [9,22]. Like other disciplines, physical education in school contributes to the general and specific training of the individual and the development of the person [9], so that they often show a more satisfactory academic performance [[23], [24], [25]], but it is the only discipline in which, through a medical certificate, some students think they can evade [26]. Adolescents with obesity escape disciplinary measures with circumvention strategies development, as in the case of anorexics. In other words, the school configuration does not allow the adolescent to break with family arrangements [26].

The physical education teacher helps students with physical disabilities to succeed in social integration [27,28]. In fact, adopting the culture of empathy promotes a school climate not only conducive to learning but also to interpretative plurality, the only way to guard against the risks of dogmatic positions that never consider the other as a possible version of themselves [9]. On the other hand, physical education is often experienced as torture, in particular with repeated failures reinforcing the feeling of ignorance and incompetence [29]. Furthermore, students with obesity need to develop skills and participate in activities just like their peers in an environment that accommodates their motor abilities while ensuring their safety [20]. Thus, the presence of students with overweight and obesity becomes a great problem for the teacher [28]. However, few studies concerning field actions are aimed at dealing with this problem.

Some studies showed that intrinsic motivation, satisfaction, and physical enjoyment improved physical performance [30,31]. Physical enjoyment can also be described as an affective and emotional reaction to a physical education session that could generate a certain satisfaction, pleasure self-confidence, and more satisfactory emotion management during physical education and sport, in children and adolescents with and without disability [[25], [32], [33], [34]].

Previous research found that girls with obesity are less likely to enjoy physical education when compared to boys, that may be because girls have on average, lower perceived physical ability, or unmotivated behaviors [35,36]. However, adapted physical activity interventions could reduce this gap of overweight girls. Therefore, the objective of this study was first to investigate the different physical enjoyment among overweight girls and boys, during adapted physical education sessions.

In addition, a gap exists in the literature regarding the effects of adapted physical exercises on the perception of physical difficulty in obese students; we hypothesized that adapted physical education sessions would lead to a decrease in perceived difficulty and an increase in physical enjoyment among overweight adolescents who participate in basketball. By addressing this knowledge gap, we aim to provide insights into effective intervention strategies to promote physical activity and well-being among overweight youth in educational settings.

## 2 Materials and methods

### 2.1 Participants

A total of 66 students with overweight participated in this study. These students were enrolled in the 1st year and 2 nd year classes of secondary school in Regueb (Tunisia). Participants were randomly assigned to an experimental group (EG; n = 32; 20 boys and 12 girls) and a control group (CG; n = 34; 21 boys and 13 girls). All measurements are represented in Table 1.

**Table 1:** Baseline participant characteristics.

Gender	Group	N	Age (years)	Height (m)	Weight (Kg)	BMI (kg/m^2^)
**Girls**	EG	20	16.00 ± 0.86	1.57 ± 0.47	67.12 ± 3.93	27.33 ± 1.53
	CG	21	16.14 ± 1.01	1.59 ± 0.06	67.95 ± 5.02	27.03 ± 1.49
**Boys**	EG	12	16.17 ± 0.58	1.66 ± 0.06	73.06 ± 3.83	26.00 ± 1.18
	CG	13	16.08 ± 0.80	1.63 ± 0.06	73.15 ± 5.11	27.45 ± 1.19

BMI: body mass index; EG, experimental group; CG, control group.

Height (m) and weight (kg) were measured with a standing stadiometer (Seca 206, Homberg, Germany) and a Tanita electronic scale BT-681 W (Tokyo, Japan).

Body mass index (BMI) was calculated using the following formula: Body mass/Height^2^ (kg/m^2^). Participants were classified as overweight or obese according to the threshold definition (cut-offs) proposed by the International Obesity Task Force [30]. The study received the agreement of the management of the high schools, and the endorsement of the teachers and students concerned.

### 2.2 Recruitment and sampling procedures

Recruitment methods involved the intentional selection of participants from rural high schools over six months, from January to June 2023. Various strategies were employed, including online advertisements on platforms such as Facebook and Twitter, as well as direct visits to educational institutions to obtain approval from administrators and teachers. Sampling procedures targeted first and second-year high school students in Regueb, Tunisia, based on predefined criteria of BMI corresponding to overweight status. Randomization was then used to allocate participants into experimental and control groups. Measurements of weight, height, and BMI were taken before morning physical activities, while tests assessing physical enjoyment and perceived effort were administered after the initial and final basketball sessions. All tests were conducted during stable weather conditions to minimize external variations. Data collection was carried out in collaboration between researchers and physical education teachers, who were trained to ensure consistency in administering questionnaires on participants' perceptions of difficulty and enjoyment in physical activity.

### 2.3 Design

Seven weeks of a teaching-learning project with one 50 min session per week, which is inspired by the physical education file n°62 entitled: "Obese student in physical education: an example of partial aptitude" which was designed by the Academic Group of Versailles "Adapted physical education and physical education and Handicap" in 2004. The aim is to provide some typical physical education situations and adapt them to students with obesity.

The study was conducted in accordance with the Declaration of Helsinki for human experimentation and it has received approval by the local research ethics committee of the Higher Institute of Sport and Physical Education of Sfax (048/2022).

We used some of the exercises described to build a basketball cycle (Table 2). The methodological tool in this research was didactic engineering, proposing and then negotiating a didactic script [37]. To follow the advancement in the perception of the physical education session difficulty in adolescents with overweight during a basketball cycle based on adapted exercise and to pursue empathy in these students. Before the start of the experimental protocol, the participants were familiarized with the equipment. The sessions were assumed by the same teachers, at the same times, and on the same premises.

**Table 2:** Experimental protocol.

Pre-intervention	Intervention	Post-intervention
Difficulty perception questionnaire Physical enjoyment questionnaire	Teacher intervention through the adapted teaching/learning project. (Basketball adapted for students with obesity)	Difficulty perception questionnaire Physical enjoyment questionnaire

### 2.4 Adapted intervention: inclusivity in basketball sessions

In the initial sessions, the sessions were designed to allow adolescents to engage in enjoyable activities irrespective of their fitness levels, as the emphasis on competitiveness was minimized. As the intervention progressed, in 1v1 basketball drills, adjustments were implemented to support students with overweight during offensive play, such as limiting the defender to using one hand and delaying their involvement until after the attacker had passed the ball. Similarly, in 3v3 drills, the game format was altered to a 3v2 setup to facilitate more opportunities for attacking. Furthermore, students were allowed to rotate during offensive phases, enabling those with overweight to rest more frequently and participate effectively.

**Fig. 1:**
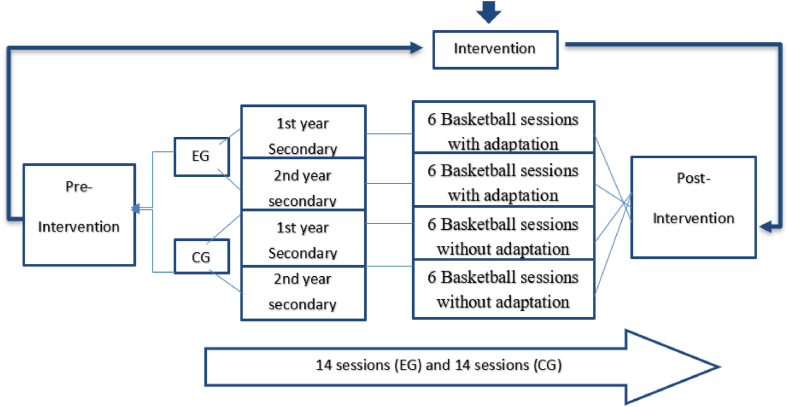
Experimental protocol. EG, Experimental group (practice basketball cycle with adaptation); CG, control group (practice classic basketball cycle).

It's also worth noting that the basketball sessions were coeducational, involving both boys and girls, as usual (Fig. 1).

Meanwhile, the students included in the control group (CG) practiced the same number of classic basketball sessions (Fig. 1).

### 2.5 The perception of difficulty (DP-15)

Before and after the adapted basketball cycle, each participant is asked to complete the scale for the perception of the difficulty of the task. The rating scale is made up of 15 levels and 7 labels, built according to the Borg Scale. The levels are numbered from 1 to 15. The rating corresponds to the label ranging from “extremely easy” to “extremely difficult” [38].

### 2.6 Physical activity enjoyment scale

This scale was used to assess the level of enjoyment following the cycle of basketball in students with obesity [39] using participants’ responses to 18 items rated on a 7-point bipolar rating scale. The assessment consists of questions relating to enjoyment after intervention with the instruction “Please rate how you feel at the moment about the physical activity you have been doing”. Overall enjoyment of physical activity score was generated by summing the individual item scores. Scores range from 18 to 126 with higher scores reflecting higher levels of enjoyment.

## 3 Statistical analysis

Data were analyzed using the Statistical SPSS 18.0 software (SPSS Inc, Chicago, Il). Values for continuous variables were expressed as the mean ± standard deviation (SD). The Kolmogorov-Smirnov test was performed to check for normal distribution. A two-way analysis of variance (ANOVA) was used to examine the effects of “group” (EG and CG), “Time” (pre- and post-cycle) on perceived difficulty and PACES scores. The post-hoc test was used to compare pre-and post-cycle scores. Also, Δ% was chosen to measure the degree of evolution of these scores. The magnitude of change expressed by Cohen's d coefficient was used to give a rigorous judgment on the differences between groups [40]. The magnitude scales were considered trivial, small, medium and large, respectively, for values of 0–0.20, >0.20 to 0.50, >0.50 to 0.80 and > 0.80 [41]. An alpha level of *p* ≤ 0.05 was set for all statistical comparisons.

## 4 Results

### 4.1 Perceived difficulty

Repeated measures revealed significant time factor and “group × time” interactions for perceived difficulty scores in girls (P < 0.001; P < 0.001) and in boys (all P < 0.01) (Table 3).

**Table 3:** Main effects of time (before/after) and group (GE/GC) and their interaction on the perception of difficulty (PD) and enjoyment of physical activity (PACES) in boys and girls with overweight and obesity.

	Factors					
	Time		Group		Interaction	
Boys	**F (1.39)**	**Ƞ**^**2**^	**F (1.39)**	**Ƞ**^**2**^	**F (1.39)**	**Ƞ**^**2**^
PD	10.43**	0.21	2.56	0.12	13.76**	0.26
PACES	202.19***	0.84	17.75***	0.31	123.48***	0.76
Girls	**F (1.23)**	**Ƞ**^**2**^	**F (1.23)**	**Ƞ**^**2**^	**F (1.23)**	**Ƞ**^**2**^
PD	7.99*	0.29	1.06	0.04	6.97*	0.23
PACES	0.01	0.00	0.07	0.003	1.89	0.08

PD, perception of difficulty; PACES: enjoyment of physical activity; *significant difference at p < 0.01; **significant difference at p < 0.001; *** significant difference at p < 0.0001.

**Fig. 2:**
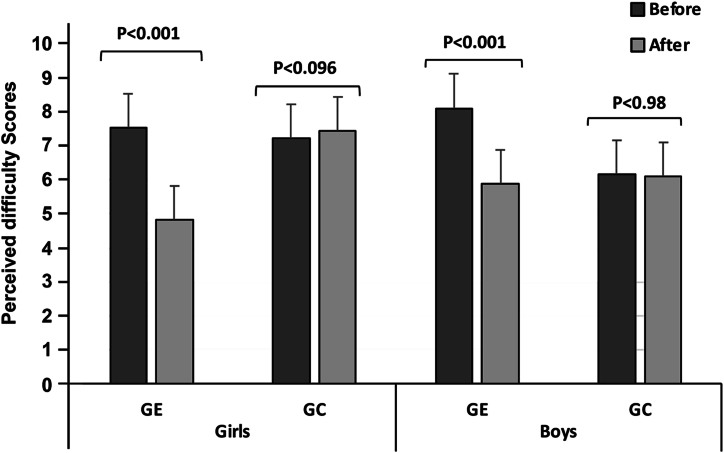
Perceived difficulties recorded before and after the basketball cycle in the experimental groups (GE) (those participating in an adapted basketball cycle) and in the control groups (GC), those participating in a traditional basketball cycle.

Statistical analysis also showed that the levels of perceived difficulty decreased significantly after the adapted basketball cycle in EG boys (P < 0.001; Δ% = - 0.27) and girls (P < 0.001; Δ% = - 0.36). There was no significant difference in GCs after the classic basketball cycle (boys: P = 0.98 and Δ% = - 0.01; girls: P = 0.96 and Δ% = + 0.03) (Fig. 2).

#### 4.1.1 Physical activity enjoyment

**Fig. 3:**
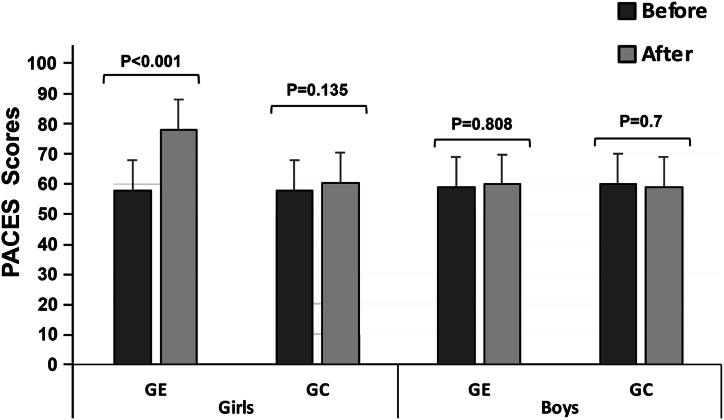
Perceived pleasures of physical activity recorded before and after the basketball cycle in the experimental groups (GE), (engaging in an adapted basketball cycle) and in the control groups (GC), (engaging in a traditional basketball cycle).

The main factors time and group and their interaction showed significant effects on PACES scores (P < 0.0001; P = 0.000; P < 0.0001) in girls with no effect on boys (Table 3). In girls, the level of enjoyment increased significantly in the EG (P < 0.001; Δ% = + 0.27) after an adapted basketball cycle. There was no significant change in the CG (Fig. 3). No effect on the level of physical enjoyment in boys has been shown (GE: P < 0.808; Δ% = + 0.01; GC: P < 0.722; Δ% = - 0.01).

## 5 Discussion

This study shows that youths with obesity had less perceived difficulties and girls reported higher physical enjoyment, after our intervention of adapted basketball, compared to peers who performed the same not adapted basketball sessions.

Overweight children and, in particular, obese adolescents experience greater psychosocial and physical discomfort (less balance and coordination, weight victimization and stigmatization and musculoskeletal pain) [42], which significantly decreases physical activity. Furthermore, low physical activity levels associate with poorer performance and low levels of several fitness components, such as muscle strength and cardiorespiratory fitness [42], which creates a vicious circle capable of worsening obesity, thus negatively impacting psycho-physical well-being [43,44]. It is difficult for obese young people to engage in physical activity, so it becomes necessary to design different approaches to physical activity, i.e. adapted to their abilities in order to encourage adherence. Many studies show that, at school level, multicomponent interventions are effective in improving eating behavior and physical activity levels of children and adolescents who are overweight or obese [7,21,25,28].

We hypothesized that a program of adapted physical activity can be useful to provide management of youth obesity, decreasing their perceived difficulty and improving physical enjoyment. Our results supported these hypotheses, demonstrating a significant reduction in perceived difficulty among participants of both sexes after the adapted basketball cycle, as well as a significant increase in physical enjoyment among girls, confirming the effectiveness of adapted physical education interventions in enhancing the experience of adolescents with overweight during physical activity while highlighting a gender difference in response. In particular, the best results obtained on girls are of significant interest, as earlier research has revealed that girls not only face more impediment to physical activity than boys [45], but that boys are more likely to experience enjoyment from physical activities [46,47].

Furthermore, although some women reported remind positive and pleasant feelings during physical activity in childhood, they were no longer evident during their adolescence [47]. Female adolescents often report negative emotions toward engaging in physical activity than male adolescents [48]. In fact, the perception of low athletic capacity [49], the feeling of embarrassment about one's physical appearance together with the negative perception of one's body represent the main obstacles to physical activity for girls [50].

As is known, physical exercise produces sensations of pleasure that depend on the participation and commitment of students in the activity carried out [51]; thus, our results substantiate antecedent research which demonstrated that enjoyment is a construct related to physical activity, enthusiasm, and involvement of students [46].

However, sensations of pleasure are inversely related to the intensity of the exercise. Feelings of high exercise intensity, perceived difficulty, and frustration influence overweight students' activity during physical education lessons [28,30]; therefore, the enjoyment experienced by children and adolescents is greater when they are taught physical education games compared to standard teaching [46]. Similarly to other studies, this research on basketball games shows a greater enjoyment levels [9,52], rendering it a more advisable type of technique confronted to conventional teaching methods that do not use games.

Our results contribute to a better understanding of potentially effective intervention since we reported that adapted basketball sessions reduced the level of perceived fatigue and improved perceived exertion and physical enjoyment in students with obesity or overweight.

However, future studies may consider the inclusion of other relevant factors and data that were not considered in this study, which represents a limitation in deeper understanding of the inclusion process of adolescent with overweight. Furthermore, data features do not allow an in-depth explanation of the reasons for the positive responses observed, such as the possible correlation with the improvement of body image problems, perhaps through a reduction in BMI due to physical education. Furthermore, it would be relevant to consider more in-depth investigations involving larger groups and different games/sports to explore responses to physical exercise tailored for different genders and ages.

Obesity could influence school integration and it is necessary to opt for adapted and motivational learning situations to improve the teaching-learning process. Therefore, physical education teachers must adapt exercises to facilitate learning and reduce the perception of effort, motivating them to commit more to physical education sessions.

These adaptations of physical education lessons are no longer a choice but a pedagogical need and necessity to improve the teaching-learning process.

## 6 Conclusion

Our results have provided specific data on the impact of adapted basketball interventions among obese adolescents, emphasizing the importance of considering gender differences in the design of these interventions. Anyway, both males and females can experience enjoyment in the same physical education interventions, albeit, at different levels of enjoyment depending on factors such as age and the specific type of activity. In fact, gender is not the unique factor capable of determining enjoyment; thus, further studies are necessary with the aim of identifying other factors able to contribute to the aforementioned enjoyment.

## Funding

This research received no external funding.

Institutional review board statement.

The study was conducted in accordance with the Declaration of Helsinki and approved by the local research ethics committee of the Higher Institute of Sport and Physical Education of Sfax (048/2022)

### Informed consent statement

Informed consent was obtained from all subjects involved in the study.

## Data availability statement

The data associated with our study have not been deposited in a publicly available repository. Data will be made available on request.

## Research ethics

We further confirm that any aspect of the work covered in this manuscript that has involved human participants has been conducted with the ethical approval of all relevant bodies and that such approvals are acknowledged within the manuscript.

## CRediT authorship contribution statement

**Oumayma Slimi:** Writing – original draft, Investigation, Formal analysis, Data curation. **Antonella Muscella:** Writing – review & editing, Supervision, Formal analysis, Conceptualization. **Santo Marsigliante:** Methodology, Investigation. **Mourad Bahloul:** Validation, Supervision, Methodology, Conceptualization.

## Declaration of competing interest

The authors declare that they have no known competing financial interests or personal relationships that could have appeared to influence the work reported in this paper.

No funding was received for this work.
